# A Law to Regulate the Practice of Dentistry in the State of New Jersey

**Published:** 1874-11

**Authors:** 


### A LAW TO REGULATE THE PRACTICE OF DENTISTRY IN THE STATE OF NEW JERSEY.

Below we append a copyof the dental law of New Jersey, and would commend special attention to it. In its provisions we regard it as the best law extant, and it is so expressed as to commend itself to the reason and good judgment of any who have or may give the matter thought. Its aim and intentions are the same as the Ohio law, but it is more explicit. Those who were in practice at and previous to the passage of the law are exempt from its requirments. The penalty is greater than the Ohio law, and the directions for its execution are more explicit. If this law is properly enforced it will bring the dental profession in New Jersey to a very enviable point of excellence. It received the signature of the Governor and became a law in full force, March 14th, 1873.

We suggest to the prfession in other states, go and do likewise.

"An Act to regulate the practice of Dentistry, and to protect the people against empiricism in relation thereto, in the State of New Jersy."

"Be it enacted *by the Senate and General Assembly of the State of New Jersey*, That from and after the passage of this act it shall be unlawful for any person to engage in the practice of dentistry in the State of New Jersey, unless such person has graduated and received a diploma from a dental college, chartered under the authority of some one of the United States or foreign governments, or shall have obtained a certificate from a board of dentists, duly authorized and appointed by this act, to issue such certificates.

*And be it enacted*, That the board of examiners shall consist of five practitioners of dentistry, who are members in good standing, of the New Jersey State Dental Society; *provided*, that said practitioners have been practicing in the state of New Jersey for a term of not less than three years; said board shall be elected by the New Jersey State Dental Society, to serve for one year; the president of said New Jersey State Dental Society shall have power to fill all vacancies in said board for unexpired terms.

*And be it enacted*, That it shall be the duty of this board, first, to meet annually at the time of meeting of the New Jersey State Dental Society, or oftener, at the call of any three of the members of said board; thirty days' notice must be given of the annual meeting; secondly, to prescribe a course of reading for those who study dentistry under private instruction; thirdly, to grant a certificate to all applicants who undergo a satisfactory examination; fourthly to keep a book in which shall be registered the names of all persons having certificates to practice in the state of New Jersey, after the passage of this act.

*And be it enacted*, That the book so kept, shall be a book of record, and a transcript from it, certified to by the officer who has it in keeping, with the common seal, shall be evidence in any court in the state.

*And be it enacted*, That three members of said board shall constitute a quorum for the transaction of business, and should a quorum not be present on the day appointed for their meeting, those present may adjourn from day to day until a quorum is present.

*And be it enacted*, That any person who shall, in violation of this act, practice dentistry in the state of New Jersey for fee or reward, shall be liable to indictment, and on conviction, shall be fined not less than fifty, or more than three hundred dollars; *provided*, that nothing in this act shall be construed to prevent any person from extracting teeth; *and provided further*, that none of the provisions of this act shall apply to regular licensed physicians and surgeons.

*And be it enacted*, That on trial of such indictment it shall be incumbent on the part of the defendant to show that he has authority, under the law, to practice dentistry to exempt himself from such penalty.

*And be it enacted*, That one half of all fines collected shall inure to the informer and the other half to the educational fund of the county.

*And be it enacted*, That nothing in this act shall apply to persons who shall be engaged in the practice of dentistry in this state at the time of the passage of this act.

*And be it enacted*, That to provide a fund to carry out the provisions of the third section of this act, it shall be the duty of the board of examiners to collect from all who receive the certificate to practice dentistry, the sum of thirty dollars each, of which sum, if there be any remaining after liquidating necessary expenses, the balance shall be paid into the treasury of the said New Jersey State Dental Society, to be kept as a fund for the more perfect carrying out of the provisions of this act; and the board of examiners for their remuneration shall receive from the above fund ten dollars per day for each day of actual service.

*And be it enacted*, That this act shall take effect immdiately."

